# Resveratrol, cancer and cancer stem cells: A review on past to future

**DOI:** 10.1016/j.crfs.2020.10.004

**Published:** 2020-11-24

**Authors:** Vasanth K. Bhaskara, Bharti Mittal, Vijaya V. Mysorekar, Nagarathna Amaresh, Jesus Simal-Gandara

**Affiliations:** a Department of Biochemistry-PG, Ramaiah Post Graduate Center, Ramaiah College - RCASC, Bengaluru 560054, India; b Immuniteit Lab Pvt Ltd., Electronic City, Bengaluru 560024, India; c Department of Pathology, Ramaiah Medical College & Hospitals (RMCH), Bengaluru 560054, India; d Department of Biotechnology, Ramaiah Post Graduate Center, Ramaiah College – RCASC, Bengaluru 560054, India; e Nutrition and Bromatology Group, Department of Analytical and Food Chemistry, Faculty of Food Science and Technology, University of Vigo – Ourense Campus, E32004 Ourense, Spain

**Keywords:** Resveratrol, Cancer, Cancer stem cells, Therapeutic targeting, Signal transduction, Resistance, *In vitro* and *in vivo* studies

## Abstract

Cancer remains to be an unresolved medical challenge despite of tremendous advancement in basic science research and clinical medicine. One of the major limitations is due to the side effects of chemotherapy which remains to be palliative without offering any permanent cure for cancer. Cancer stem cells (CSCs) are the subpopulation of cells in tumors that remain viable even after surgery, chemo- and radio-therapy that eventually responsible for tumor relapse. Hence, by eliminating non-stem cancer cells and cancer stem cells from the patient, permanent cure is expected. Phytochemicals have been under the intensive study to target these CSCs effectively and permanently as they do not cause any side effects. Resveratrol (RSV) is one such compound attaining lot of interest in recent days to target CSCs either alone or in combination. RSV has been used by several researchers to target cancer cells in a variety of disease models, however its CSC targeting abilities are under intensive study at present. This review is to summarize the effects of RSV under *in vitro* and *in vivo* conditions along with advantages and disadvantages of its uses against cancer cells and cancer stem cells. From the first reports on phytochemical applications against cancer and cancer stem cells in 1997 and 2002 respectively followed by later reports, up to date observations and developments are enlisted from PubMed in this comprehensive review. RSV is shown to be a potential compound having impact on altering the signal transduction pathways in cancer cells. However, the effects are variable under *in vitro* and *in vivo* conditions, and also with its use alone or in combination with other small molecules. Past research on RSV is emphasizing the importance of *in vivo* experimental models and clinical trials with different prospective combinations, is a hope for future promising treatment regimen.

## Highlights

• Resveratrol is a potent reducing agent, and can prevent carcinogenesis due to its anti-oxidant abilities
• There is effect of resveratrol with long noncoding RNAs (lncRNAs) in lung carcinogenesis
• It acts as an immunomodulatory agent for treating cancer
• The major drawback of is that require validation of the same effect under *in vivo* conditions
• It should be tested with different combinations as can affect multiple pathways unlike targeted drugs

## Graphical abstract

**Figure:**
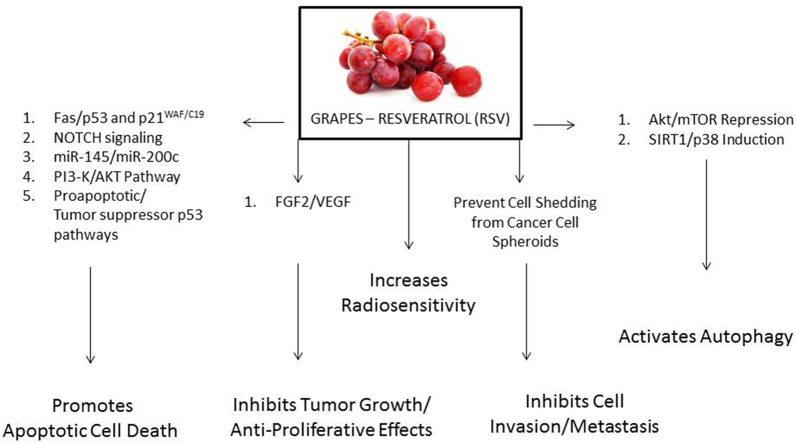

## Acronyms list

ABC: ATP-binding cassette transporters
ALDH: Aldehyde dehydrogenase
AML: Acute myeloid leukemia cells
AT/RT: Atypical teratoid/rhabdoid tumor
Bax: Bcl2 associated X protein
Bcl2: B-cell lymphoma-2
BNIP3: BCL2/adenovirus E1B 19 kDa protein-interacting protein-3
CD: Cluster of differentiation
CDK: Cyclin-dependent kinase
CHD: Coronary heart disease
COX: Cyclooxygenase
CSCs: Cancer stem cells
CYP: Cytochrome P450
DAPK2: Death associated protein kinase-2
EMT: Epithelial to mesenchymal transition
eNOS: Endothelial nitric oxide synthase
ER: Estrogen receptor
ERK: Extracellular signal regulated kinase
ESA: Excretory secretory antigen
FA: Fanconi anemia
FAS: Fatty acid synthase
GBM: Glioblastoma multiforme
HER-2: Human epidermal receptor-2
HIF-1α: Hypoxia inducible factor -1α
IL: Interleukins
iNOS: Inducible nitric oxide synthase
JAK: Janus kinase
lncRNA: Long non-coding RNA
ALT-1: Mucosa-associated lymphoid tissue lymphoma translocation protein
MAP: Mitogen activated protein kinase
MCP-1: Monocyte chemoattractant protein-1
MDR1: Multi-drug resistance protein-1
MEK: Mitogen activated protein kinase - MAPK Kinase
MMP: Matrix metallo proteinase
MRP1: Multidrug resistance associated protein-1
mTOR: Mammalian target of rapamycin
NAC: N-acetyl cysteine
NF-κB: Nuclear factor kappa B
nNOS: Neuronal nitric oxide synthase
NO: Nitric oxide
Nrf-2: Nuclear factor erythroid-2 related factor-2
ODD: Ornithine decarboxylase
PI3K: Phosphoinositide 3-kinase
PPAR: Peroxisome proliferator-activated receptor
QR2: Quinone reductase-2
RAF: Rapidly accelerated fibrosarcoma protein kinase
RAS: Rat sarcoma protein kinase
RCC: Renal cell carcinoma
ROS: Reactive oxygen species
RSV: Resveratrol
SCC: Squamous carcinoma cell
SERM: Selective estrogen receptor modulator
SIRT1: NAD-dependent deacetylase sirtuin-1
SREBP1: Sterol regulatory element binding protein-1
STAT: Signal transducer and activator of transcription
TGF: Transforming growth factor
TNBC: Triple negative breast cancer
TRAIL: Tumor necrosis factor related apoptosis inducing ligand
TrxR: Thioredoxin reductase
VEGF: Vascular endothelial growth factor

## 1 Introduction

Resveratrol (RSV), is 3,4’,5 – trihydroxy stilbene, a phytoalexin is widely distributed in variety of plants including red grapes, berries, peanuts, etc. Highest levels of RSV are found in Japanese knotweed (*Polygonum cuspidatum*) and muscadine grapes (*Vitis rotundifolia*) (Shrikanta et al., 2015). Though its occurrence is widely distributed about more than 70 plant species, its bioavailability is challenging upon its consumption (Gambini et al., 2015). Tome-Carneiro et al. (2013) have further shown, different levels of RSV concentrations are attributed for differential health impacts. Szekeres et al. (2010) in their review demonstrated that, due to the presence of three hydroxyl groups, it was known to act as a potent anti-oxidant by interfering with intracellular redox signaling. In many studies with different model organisms, RSV is shown to increase healthy life span mediated by SIRT1 (NAD-dependent deacetylase sirtuin-1) (Bhullar and Hubbard, 2015). RSV can reduce inflammatory stress through its effects on mitochondria. It activates a group of mitochondrial proteins of sirtuin family, particularly SIRT1. Lagouge et al. (2006) had shown that activation of sirtuin family protein can in turn related to the blood sugar stabilization in the body.

RSV effects on nitric oxide cycle were well known, through which it maintains the health of immune, nervous and vascular system. Nitric oxide in the body is synthesized by the enzyme Nitric Oxide Synthase (NOS) which has a critical role in inflammation. NOS can occur in different isoforms based on its location such as endothelial NOS (eNOS), neuronal NOS (nNOS) and inducible NOS (iNOS). All the NOS isoforms (eNOS, nNOS and iNOS) have been reported to be expressed in the cardiac and endothelial cells of the blood vasculature. RSV has been proved to show its effects by acting on eNOS derived NO system thus inhibiting the damage caused due to stress-induced inflammation (Xia et al., 2014). These effects are well established functions of RSV on cardiac health. However, RSV has also been shown to exhibit broad-spectrum antimicrobial, anti-infective, anti-amyloidogenic activities and now researchers are testing for the efficacy of its anti-cancer stem cell properties.

This review is a comprehensive collection of original work and reviews to elucidate the present idea about advantage of resveratrol application particularly against CSCs. This review is also to discuss about *in vitro* and *in vivo* observations of RSV effects emphasizing its efficacy to use in future cancer therapy.

## 2 Resveratrol mechanisms affecting cancer cells

In the recent past, a lot of interest has been aroused in revealing the exact mechanisms of anti-cancer effects of RSV. It is a polyphenolic stilbene with an aromatic benzene bonded to three hydroxyl groups that acts as a potent anti-oxidant neutralizing the toxic effects of reactive oxygen species (ROS) in the body, thereby neoplastic transformation of cells can be prevented. However, the anti-cancer effects have been reported due to other mechanism of action as its anti-oxidant potential is not very high when compared with other biological molecules. RSV has been reported to exert its anti-cancer activity by inducing cell cycle arrest, apoptosis, differentiation and inhibiting cancer cell proliferation. Jang et al. (1997) for the first time evidenced that from topical application of RSV in an experimental skin cancer mouse model tumorigenesis found to be inhibited. RSV is shown to be effective by acting at initiation, progression and metastasis stages of tumorigenesis (Ko et al., 2017).

**Fig. 1:**
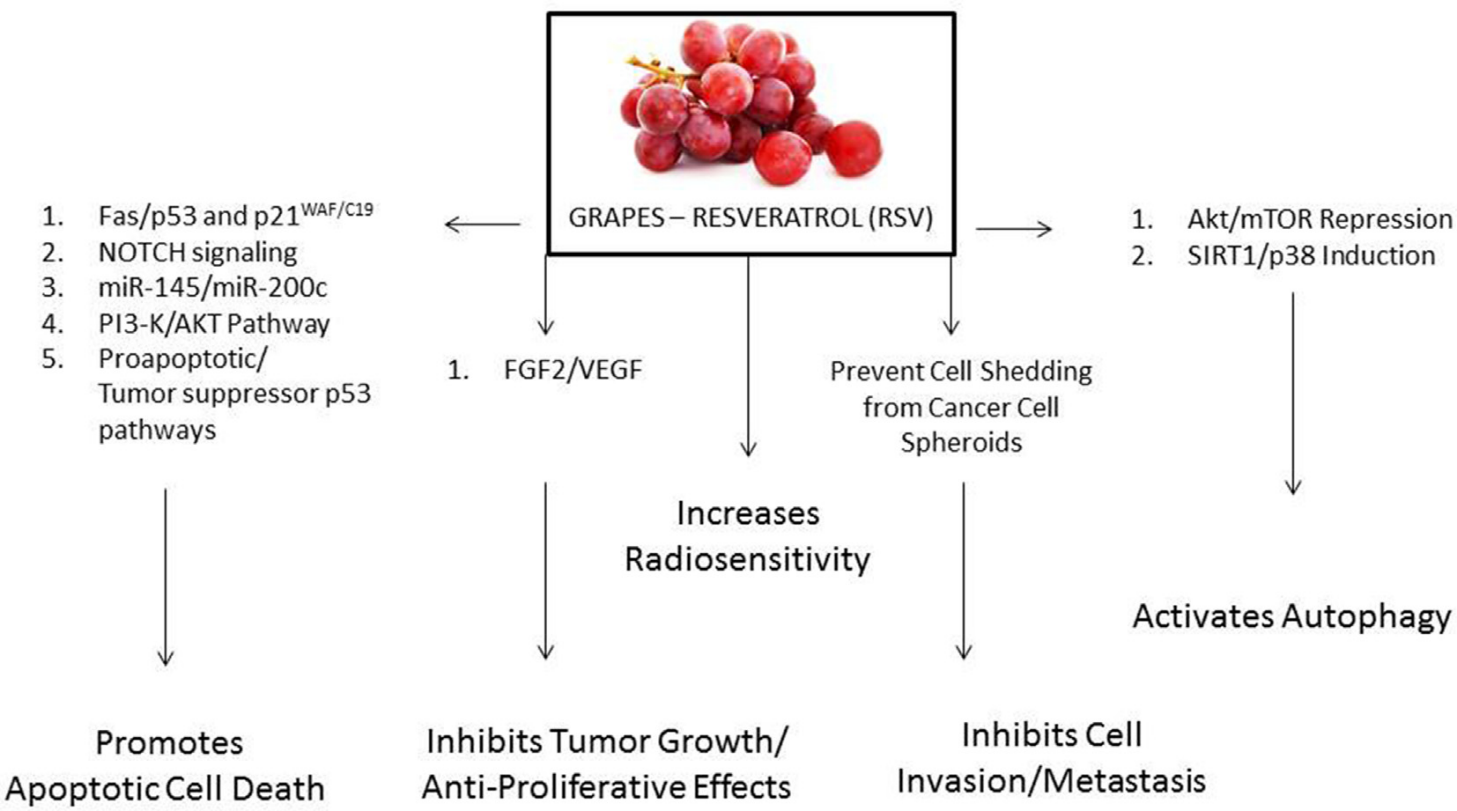
Resveratrol effects on cellular pathways and its mediated anti-cancer effects.

**Fig. 2:**
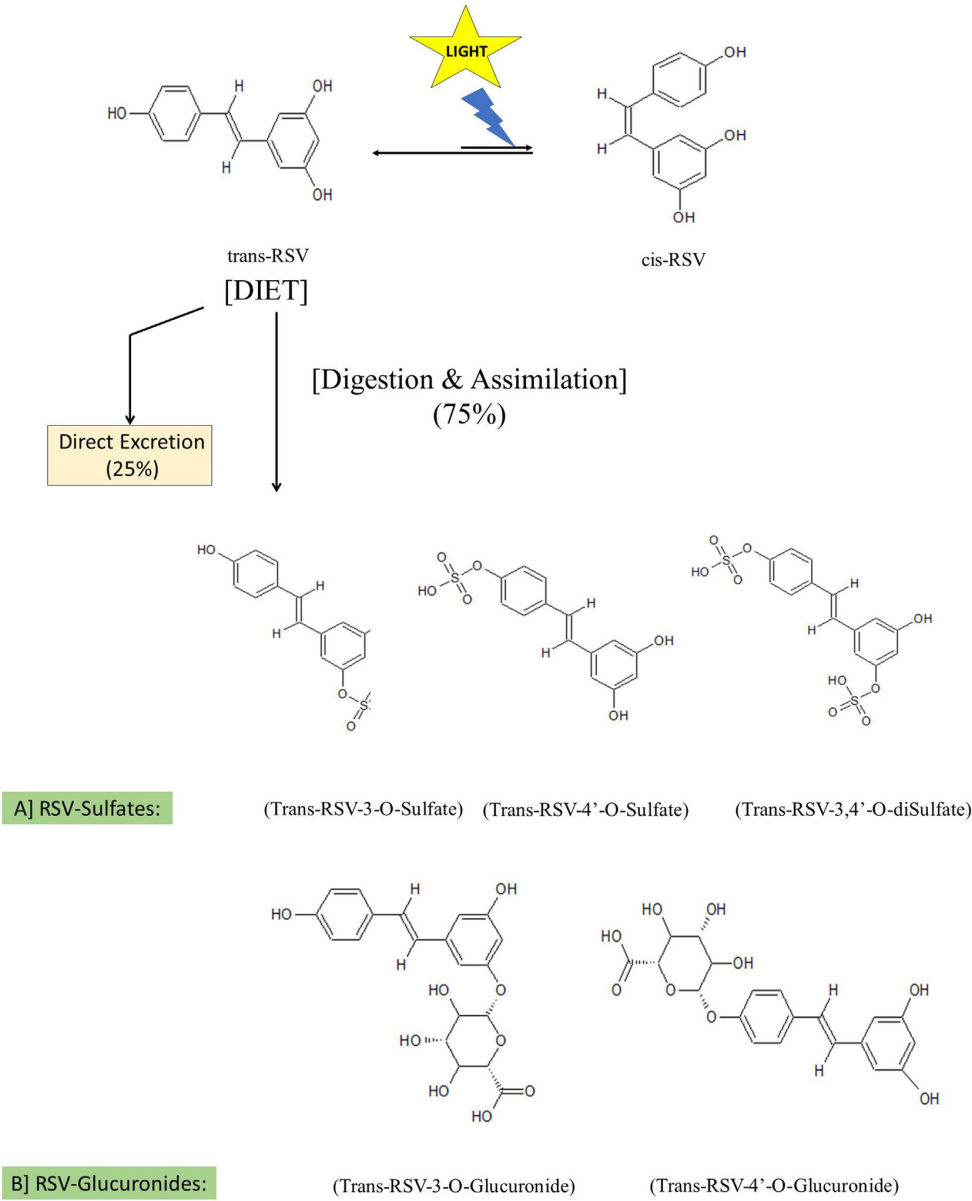
Resveratrol isomers & its immediate metabolites.

There are myriad pathways that RSV has been shown to influence on cancer cells. However, these effects are observed to be limited by the experimental conditions. Still it requires significant efforts to identify cross-talk pathway effects and to select the common key targets in cancer cells. Fig. 1, Fig. 2.

### 2.1 Resveratrol structure and anti-cancerous activity relevance

Resveratrol is 5-[(E)-2-(4-hydroxyphenyl)ethyl]benzene-1,3-diol with three hydroxyl groups attached to carbon atom of the two aromatic ring structures. In plants it is synthesized to provide protection against the environmental stress and to a variety of infections. The therapeutic effects of resveratrol originally started from the concept of “French paradox”, which came into an existence in the year 1992 in an epidemiological study by Renaud and de Lorgeril (1992), to understand the effect of wine consumption on coronary heart disease (CHD). Later studies have characterized different compounds in red wine with a variety of flavanols like myricetin, kaempferol, quercetin (predominant), catechin, epicatechin, oligo- and poly-meric flavan-3-ols, proanthocyanins, anthocyanins, phenolic acids such as gallic acid, caftaric acid, caffeic acid, p-coumaric acid and the resveratrol (stilbene).

The compounds with polyphenolic substituents particularly catechols or 1,4 dihydroquinone are unique in forming stable phenoxyl radical upon reaction with oxidizing agents like superoxide radicals, peroxynitrite, etc., formed in the cells posed under oxidative stress. As wine was found to be enriched with catechols, its effects initially were explained for anti-fungal, potential anti-platelet aggregation and anti-oxidant properties (Waterhouse, 2002). RSV accumulation in grape plants was found to be formed in response to *Botrytis cinerea* and other fungal infections. In the plants, oligomers of RSV are known as viniferins that are actual anti-fungal compounds. In wine, *cis-*, *trans-* and glucosides of both *cis-* and *trans*-resveratrol are found. Trela and Waterhouse (1996) have observed that in plants, *cis-*resveratrol is absent and is formed in wine due to light induced *cis/trans* isomerization. In dietary products, RSV occurs in glycosylated form known as “piceid” which is resistant to undergo enzyme mediated oxidation, thereby retaining its biological effects. However, intestinal cells absorb free form of RSV after the action of glycosidases there by enzyme activity is related to the absorption of RSV into the body (Fan et al., 2009).

Resveratrol in wine was explained as an important derivative of red grapes and as a constituent of biological fluid that could prevent tumor growth for the first time in 1997. Due to its structural similarity with that of the diethylstilbestrol which is a synthetic estrogen, RSV was considered as a phytoestrogen. RSV was found to inhibit the binding of radio-labeled estradiol to estrogen receptor responsible for variable effects under different test systems relating its influence on breast cancer (Gehm et al., 1997). It was reported that RSV has 16 times lower anti-oxidant potential than the α-tocopherol. But unlike other polyphenol molecules, RSV can undergo redox cycling being able to adopt quinone like structure. ROS production in the cell reported to cause activation of nuclear factor erythroid-2 related factor-2 (Nrf-2) which regulates the oxidative stress. Further, it was shown to improve recycling and cross-talk interactions with central and lipid metabolism along with modulating phase-I and II metabolism enzymes and transporters. Quinone reductase-2 (QR2), a phase-II detoxifying enzyme shown to interact directly with RSV. Inhibition of QR2 by RSV can induce other cellular anti-oxidant enzymes and thus increases cellular resistance to oxidative stress (Britton et al., 2015).

It has been shown that several methylated compounds generate formaldehyde in the cell, which was shown to be effectively being prevented by RSV. Further, the reaction products formed during the interaction between RSV and formaldehyde can act as chemopreventive factors (Tyihak et al., 1998). Thus Szende et al. (1998) reported that RSV, due to its formaldehyde capturing ability can influence cell proliferation and active cell death in a dose-dependent manner. Further, Fontecave et al. (1998) also shown that RSV can act as a potent inhibitor of ribonucleotide reductase and DNA synthesis in mammalian cells thereby controlling the cell proliferation and exhibiting chemopreventive activity.

After ingestion, RSV undergoes a variety of biotransformation in the intestinal cells, liver cells and then by the gut microbiome. From the diet, about 75% of RSV gets assimilated and metabolized rapidly and extensively to form conjugated products. Remaining 25% of ingested RSV will be excreted directly through urine. About 2% of RSV in plasma can be regenerated from the conjugated metabolites upon hydrolysis by the enzymes of microbiome. Though some biological effects have been shown to exert by RSV metabolites, in the literature the anti-cancer effects are mainly attributed only for the free form of RSV (Springer and Moco, 2019).

### 2.2 Anti-proliferative effects

Cancer cell proliferation is attributed due to an aberrant Mitogen Activated Protein (MAP) kinase signaling pathways. Constitutive activation of RAS/RAF/MEK/ERK (extra-cellular signal regulated kinase) pathway has significant role in the sustained cancer cell survival and proliferation. In different cancers, alterations mostly may occur at the receptor level or due to consecutively mutated downstream kinases of the respective pathways. For instance, in pancreatic cancers, epidermal growth factor (EGF) and/or human epidermal receptor-2 (HER-2) mutations are critically responsible for cancer cell proliferation (Oliverira-Cunha et al., 2011). Further, cancer cells secrete vascular endothelial growth factors (VEGF) that can induce neovascularization and that may in turn provoke cell proliferation. In renal cell carcinoma cells (RCCs) including ACHN and A498, RSV treatment is found to exert its effect on RCC proliferation, migration and invasion in a concentration dependent manner through inactivation of the Akt and ERK1/2 signaling pathways (Zhao et al., 2018). RSV is observed to have effect on VEGF expression mediated regulation of cell proliferation under *in vitro* condition (Liu et al., 2012). In CaCo-2 cells, treatment with 25 μM RSV has shown 70% growth inhibition due to S/G2 phase arrest. These effects were shown to be due to the inhibition of ornithine decarboxylase (ODD) activity which is enhanced in cancer cells (Schneider et al., 2000).

### 2.3 Cell-cycle arrest and pro-apoptotic effects

RSV is reported to be responsible for cell cycle arrest thereby inducing the cancer cells to undergo apoptosis. Singh et al. (2017) reported that the combined drug treatment of RSV and docetaxel on C4–2B and DU-145 cell lines of prostate cancer was found to be responsible for inhibited progression of G2/M phase arrest and also enhanced expression of pro-apoptotic genes of *Bax*, *Bid* and *Bak*. In an another study, Yuan et al. (2015) reported that A549 cells of lung cancer, upon treatment with RSV was found to arrest the cell cycle in G_0_/G_1_ phase by down regulating the expression levels of cyclin D1, cyclin-dependent kinase-4 (CDK4) and CDK6 along with an upregulated expression of CDK inhibitors, p21 and p27 in a p53 independent manner. Mitochondria in the cell have a very critical role in normal cells to decide cell survival and death fates through maintenance of an optimal Bcl2/Bax ratio. Kumar et al. (2017) shown that RSV treatment has resulted in decreased cell viability, altered cell morphology and increased apoptosis in a dose, time and caspase-independent manners in murine prostate cancer. These effects were due to the influence of the RSV via disrupted mitochondrial membrane potential and aberrant expression of Bax/Bcl-2 proteins.

### 2.4 Anti-metastatic effects

Metastasis is the later event of tumor progression that causes seeding of tumor cells in distant metastatic sites ultimately leading to the formation of secondary tumors. De-differentiation of cancer cells in later stages that gets induced by tumor microenvironment has been reported to be associated with enhanced metastatic abilities of cancer cells due to acquired stemness (Quail and Joyce, 2013). Cancer stemness is known to enhance the metastatic potential of several cancer types leading to aggressive secondary tumor formation at different sites (Lif et al., 2007; Li and Li, 2014). Ji et al. (2013) reported in an *in vitro* study, RSV treatment lead to inhibited invasion and metastasis of colorectal cancer-derived cell lines LoVo and HCT116 by suppressing the Wnt/β-catenin signaling mediated target genes of c-Myc, MMP-7, and MALT-1. At low doses, RSV is shown to be effective against breast cancer metastasis to lungs in mice by its inhibitory effect on Stat3 mediated signaling (Lee-Chang et al., 2013). The metastasis of 4T1 mouse breast cancer cells both under *in vitro* and *in vivo* conditions upon RSV pre-treatment was found to inhibit cancer cell metastasis through its inhibitory effect on MMP-9 expression (Lee et al., 2012).

## 3 Strategies to eliminate cancer stem cells

Most of the tumor tissues types were first discovered to contain heterogeneous cell population with distinct levels of therapeutic resistance, self-renewal capacities, low-proliferation rate and with the ability to repopulate original tumor cells (Chang, 2016). Further, these populations of cells were named as cancer stem cells, which are responsible for chemo resistance and tumor relapse (Nguyen et al., 2012). It has been concluded from different reports, that by eliminating cancer stem cells completely from the tumor site, permanent cure for cancer can be achieved. Hence, targeting the minor cancer stem cell population is a very important and prospective strategy of cancer treatment.

There have been different strategies in the literature to eliminate these CSCs such as inducing CSC differentiation and then targeting by potent apoptotic inducers, by targeting DNA damage repair enzymes, by targeting cell cycle specific regulators, by using monoclonal antibodies, by altering the drug resistance genes and recently by metabolism based therapeutic targeting. These strategies are dependent on the cancer type, specific to the stage and based on experimental system under the study (Yoshida and Saya, 2016; Jagust et al., 2019; Shibata and Hoque, 2019). RSV is reported to regulate all the major CSC signaling pathways, but exact mechanisms of its interactions are not clearly understood (Zhang et al., 2018). In spite of the promising results of CSC targeting under *in vitro* conditions, it require robust research to translate the observations to *in vivo* systems and further to the clinical settings.

### 3.1 Therapeutic resistance of cancer stem cells and resveratrol

Cancer stem cells are described to exhibit endogenous resistance mechanisms against radiation and chemotherapy due to preferential activation of DNA damage response, hypoxic stability, an increased activity of ABC transporters leading to efficient drug efflux, elevated expression of anti-apoptotic molecules, higher aldehyde dehydrogenase (ALDH) activity/enhanced activity of repair enzymes and quiescence or dormancy or Go – Phase (Prieto-Vila et al., 2017; Cho and Kim, 2020). Resveratrol has been shown to reverse the resistance to standard classical chemotherapeutics in non-stem cancer and cancer stem cells by sensitizing the cells in multiple ways. It is reported to cause an increased susceptibility to induce cancer cell apoptosis by interfering with pro- and anti-apoptotic factors, by regulating miRNAs, by its effect on drug- and carcinogen-metabolizing enzymes, by interfering with drug resistance gene/protein expressions and respective signaling pathways through poorly understood mechanisms (Mieszala et al., 2018; Zhang et al., 2019).

Kao et al., (2009) and Lu et al., (2009) have reported that effects of radiotherapy in RSV pretreated medulloblastoma (MB) cancer stem-like cell cultures and CD133 – positive cells derived from atypical teratoid/rhabdoid tumor (AT/RT) was reported to be significantly enhanced. Radiation combined with RSV pretreatment was observed to significantly increase the radiosensitivity in MB-CSCs. Similarly, AT/RT-CD133 (+) cells with CSC properties when treated with RSV, reported to inhibit expression of drug resistant genes and induced differentiation of AT/RT CD133(+) cells to drug-sensitive CD133(−) cells. RSV was reported to induce chemosensitization to 5-fluorouracil through inhibition of epithelial-mesenchymal transition (EMT) factors and down regulation of NF-κB regulated (inhibited IκBα kinase and IκBα phosphorylation and degradation) gene products like MMP-9, caspase-3 in colorectal cancer cells (Buhrmann et al., 2015). Choi et al. (2016) reported that RSV analog HS-1793, found to enhance radiosensitivity in mouse-derived breast cancer cells under hypoxic conditions through inhibiting the hypoxia-inducible factor-1α (HIF-1α) and VEGF protein in FM3A mouse mammary carcinoma cells. Tumor necrosis factor related apoptosis-inducing ligand (TRAIL) armed oncolytic adenovirus known as ZD55-TRAIL, reported to enhance A549 sphere cell apoptosis through mitochondrial pathway up on treatment of RSV along with small molecules embelin and LY294002 and thus shown an improved survival status of lung cancer mouse models (Yang et al., 2015a, b, c). Zhou et al. (2019) reported an increased chemotherapeutic response by RSV pretreatment which has reversed the stemness induced by gemcitabine in pancreatic cancer cells of MiaPaCa-2 and Panc-1 cells via targeting sterol regulatory element binding protein-1 (SREBP1). In SKOV3 - cancer stem cells of ovarian cancer, RSV found to potentially increase the tumoricidal effect of chemotherapeutic doxorubicin under *in vitro* conditions (Pouyafar et al., 2019a, Pouyafar et al., 2019b). Though couple of studies indicated role of RSV in reversing the cancer stem cell drug resistance, its mechanism of intervention has to be understood in detail in *in vivo* models and in human trials.

### 3.2 Natural products strategy

Recently, there has been a lot of attention on natural dietary product characterization with medicinal properties that can control cancer cells preventing their progression. Further, these compounds have attained importance in research and drug discovery due to their less or no toxic side effects (Rajesh et al., 2015). Panche et al. (2016) had discussed in detail about the current trends of research and developments on flavonoids as potential drug candidates. Different chemical ingredients in the diet consumed in day-to-day life have been studied for their potential benefits. Newman and Cragg (2016) have reported that from the year 1981–2006, nearly 63% of anticancer drugs used have been developed from natural products. Applications of these natural products are shown to be particularly important in cancer therapy as they do not pose any side effects.

### 3.3 Resveratrol strategy

RSV is one of the natural products, which was known to be responsible for cardiac health and now the same RSV has generated a lot of interest for its anti-cancerous effects. Jang et al. (1997) reported for the first time that the RSV's anticancer effect was due to its anti-initiation, anti-promotion and anti-progression activities.

RSV was reported to exhibit selective estrogen receptor modulator (SERM) activity and this observation further laid possibility of its role in breast cancers (Gehm et al., 1997). Gunther et al. (2007) found that RSV can also be used to target CSCs by observing in an attempt to test the polyphenols including RSV that could prevent the cell shedding from mouse mammary cancer spheroids inhibiting the cancer cell invasion of embryonic stem cell cultures. However, Wallenborg et al. (2009) reported that by using small amount of red wine (1–5%) containing RSV exhibited massive cell death of various cell types including neural stem cells had taken place due to increased oxidative stress mediated inhibition of thioredoxin reductase (TrxR) activity but not due to RSV. Resveratrol was shown to exert effect by the down regulation of *fatty acid synthase (FAS)* gene and up-regulation of pro-apoptotic genes like DAPK2 and BNIP3 in cancer stem – like cells (CD24(−)/CD44(+)/ESA(+) which were isolated from both ER+ and ER-breast cancer cell lines. These alterations were observed to cause inhibited cell viability and mammosphere formation along with induced pro-apoptotic effects (Pandey et al., 2011). There are total 160 results have been displayed which are relevant to resveratrol and cancer stem cells in the PubMed search. It is also interesting to note that, from the year 2015 there has been increasing number of reports in the same field of research. Though majority of the attempts made were with *in vitro* model systems, many experiments were also reported by using *in vivo* models. Differential effects of RSV observed in various *in vitro* and *in vivo* cancer stem cell models have been presented in Table – 1*.*

**Table 1:** Summary of historical review of RSV effects in various CSC model systems reported.

Ref.	CSC model system	RSV Effects
Gunther et al. (2007)	4T1 Mouse mammary breast cancer cells.	Cell shedding from mouse mammary cancer spheroids **↓** Cancer cell invasion in embryonic stem cell cultures **↓**
Kao et al. (2009)	CD133-positive/negative cells derived from atypical teratoid/rhabdoid tumors (AT/RT-CD133(±)).	*With* 200 μM *treatment; in vitro* proliferation and *in vivo* tumor relapse of CD133(+) cells **↓** With 150 μM treatment; Drug resistance genes in CD133(+) cells **↓** Differentiation of CD133(+) cells into CD133(−)**↑**
Lu et al. (2009)	Medulloblastoma (MB)-associated 3D-spheroid forming CSCs	Proliferation and Tumorigenicity of MB-CSCs **↓** Radiosensitivity **↑**
Shankar et al. (2011)	Human pancreatic Cancer Stem Cells (CD133+, CD44^+^, CD24^+^, ESA+) of NOD/SCID mice, CSCs from Kras^G12D^ transgenic mice and human pancreatic tumor derived CSCs.	Caspase 3/7 **↑** Expression of XIAP, BCL-2 and CCND1 **↓**
Pandey et al. (2011)	CD24(−)/CD44(+)/ESA(+) cells from estrogen receptor – ER^+^ and ER^−^ breast cancer cell lines.	Lipogenesis by modulating FAS expression **↓** Apoptosis **↑**
Hu et al. (2012a, b)	Human promyelocytic leukemia stem cells (KG-1a)	KG-1a cells susceptible to cytokine-induced killer cell (CIK) mediated cytolysis **↑**
Hu et al. (2012a, b)	CD44 positive head and neck cancer (HNC) cells; HNC-Tumor Initiating Cells (TNCs)	Trans-differentiation of head and neck cancer-derived tumor-initiating cells (HNC-TICs) **↑** EMT **↓**
Hagiwara et al. (2012)	Orthotopic inoculation of female SCID mice with MDA-MB-231-luc-D3H2LN cells in pretreated mice with resveratrol.	Tumor suppressive miR-141 and miR-200c expression **↑** CSC phenotype **↓**
Sato et al. (2013)	Patient-derived Glioma Stem Cell (GSCs) cultures and Intracranial xenograft models of GSCs	p53-Nanog axis mediated Differentiation of GSCs **↑**
Su et al. (2013)	Human AML HL-60 cell lines and patient derived samples	Sonic hedgehog (Shh) **↓** Gli-1 nuclear translocation **↓** Cell viability **↓** IL-6 treatment induced the growth of AML cells through Shh signaling which was blocked by RSV treatment.
Sayd et al. (2014)	Glioblastoma Stem Cells (GSCs): Derived from Human glioblastoma tissue Normal Neural Stem Cells (NSCs): Derived from human fetal brain tissue	GSC proliferation **↓** up to 150 μM and necrosis **↑** at higher doses. However, it has no effect on NSCs. These effects on GSCs are mediated through Sirtuin-2 which has vital enzymatic function in tumor metabolism.
Fu et al. (2014)	Breast cancer stem-like cells (BCSCs) isolated from MCF-7 and SUM159	Administration of 100 mg/kg/day in NOD/SCID mice resulted xenograft tumors size **↓** BCSC cell population in tumors **↓** Autophagy in BCSCs **↑**
Yang et al. (2015a, b, c)	Colorectal cancer stem cells *In vitro*	Administration of 12.5–200 μ mol/L resulted in HCT116 CCSC proliferation **↓** in a dose-dependent manner.
Seino et al. (2015)	Ovarian cancer stem cells *In vitro*	**↑** Apoptosis of ovarian cancer stem cell A2780 independent of ROS **↓ S**elf-renewal capacity of A2780 stem cells depending on ROS
Clark et al. (2017)	Multiple patient-derived GBM stem-like cell (GSC) lines and established U87 glioma cells.	GBM and GSC growth and infiltration **↓** through modulation of AKT and p53
Cilibrasi et al. (2017)	Human glioblastoma tissue derived glioma stem cells (GSCs) from different patients.	Cell proliferation **↓** Cell mortality **↑** **↓** Cell motility through modulated Wnt signaling and EMT pathway mediators.
Ruiz et al. (2018)	Enriched CSCs derived from cervical cancer HeLa cell lines	RAD51 expression **↓** CD49f-positive stem cell apoptosis **↑**
Fei et al. (2018)	Malignantly transformed dendritic cell line SU3-ihDCTC induced by glioma stem cells.	*In vitro* co-cultured GSC induced malignant transformed bone marrow derived dendritic cells exhibited increased sensitivity to chemotherapeutics after RSV treatment.
Peng and Jiang (2018)	Human osteosarcoma cell lines – MNNG/HOS, MG-63 and Osteoblast line hFOB1.19.	JAK2/STAT3 **↓** Osteosarcoma cell proliferation **↓** Tumorigenesis **↓**
Song et al. (2019)	LN18 and U87glioblastoma cells; U87 xenograft models	Epithelial to mesenchymal transition (EMT) of glioblastoma cell lines LN18, U87 and U87 xenografted mice models **↓** Expression of β-catenin **↓** *GBM Stem cell marker expression:* Twist **↓** Snail**↓** Slug **↓** MMP-2 **↓** MMP-9 **↓** Smad **↓**
Buhrmann et al. (2019)	HCT116, RKO, SW480 colorectal cancer cell monolayer and 3D alginate cultures.	TNF-β/TNF-βR **↓** Epithelial-to-mesenchymal transition **↓** through NF-κB **↓** and focal adhesion kinase (FAK) **↓**
Segun et al. (2019)	Breast (MCF7), liver (HepG2), lung (A549) and prostrate (PC3) carcinoma cell lines versus normal prostrate epithelial cell (PNT2) cell lines	*Four RSV derivatives:* (E)-resveratrol 3-O-rutinoside (1), 5-methoxy-(E)-resveratrol 3-O-rutinoside (2), pinostilbene (3) and 3-hydroxy-5-methoxybenzoic acid (4) isolated from the stem bark extract of *C africana* tested for anti-cancer stem cell activities. Except the derivative – 4, all the remaining derivatives were observed to be cytotoxic across the four cell lines.
Jhaveri et al. (2019)	U-87 MG: an astrocytoma grade IV cell line and LN-18: a grade IV glioblastoma cell line neurosphere cultures	Transferrin targeted liposomal formulations of Resveratrol (Tf-RES-L) used to treat GBM neurospheres. Both free RSV and RSV-formulations were found to Anchorage-independent growth of GBM neurospheres **↓** Its action exhibited through transferrin and **↑** activated caspase – 3/7.
Zhou et al. (2019)	MiaPaCa-2 pancreatic cancer cell lines and KPC mouse models of pancreatic ductal adenocarcinoma (PDA)	Pretreatment reversed the stemness induced by gemcitabine by targeting sterol regulatory element binding protein - 1 (SREBP1) both *in vitro* and *in vivo*.
Yin et al. (2020)	Patient tissue derived ‘gastric-cancer-derived-mesenchymal stem cells – GC-MSCs	IL-6, IL-8, MCP-1,VEGF expression **↓** β-catenin nuclear translocation in GC-MSCs upon pretreatment with RSV **↓** Metastasis of GC-MSCs **↓**
Sun et al.(2020)	ACHN and 786-O derived renal carcinoma stem cells	Size and number of tumor spheres **↓** Sonic hedgehog (Shh) pathway related proteins: SHH, SMO, Gli1, Gli2 **↓** CSC marker proteins: CD44, CD133, ALDH1A1, Oct-4, Nanog **↓** Cell proliferation **↓** Apoptosis **↑**

### 3.4 Resveratrol impact on cancer stem cell signaling pathways

Cancer stemness is a spontaneous process and is mainly associated with tumor micro environmental factors that modulate the signal transduction pathways responsible for cancer stemness. The hallmark features during different types of solid tumor progression includes unregulated cell proliferation, neovascularization, hypoxia and/or intermittent hypoxia, cancer stemness and metastasis. Thus cancer stemness is presumably known to appear at the terminal stage during the tumor progression. However, there are no evidences to prove association of CSCs during the initial stages. This is another interesting area to check the stage specific effects of RSV associated with cancer stemness.

Major functional signaling pathways attributed for cancer stemness that are experimentally evidenced and are used for therapeutic targeting includes Wnt, nuclear factor-κB (NF-κB), Notch, hedgehog, janus kinase/signal transducer and activator of transcription (JAK-STAT), PI3K/AKT/mTOR (Phosphoinositide 3-Kinase/AKT/mammalian target of rapamycin), transforming growth factor (TGF)/SMAD and peroxisome proliferator-activated receptor (PPAR) pathways (Yang et al., 2020). Though some of these pathways were found to have role in cancer stemness, only anti-cancer properties of RSV were reported and its anti-cancer stemness effects are yet to be evidenced. It has been reported that in Indian triple negative breast cancers (TNBC) patients, the putative cancer stem cell marker CD133 or prominin-1 is correlated with the functional CSC signaling pathways including NOTCH-1/HES-1; Wnt/β-catenin; TGF-β III R/SMAD-7 and PTCH-1/Gli-1 (hedgehog) pathway activations (Bhaskara et al., 2019).

Phytochemicals can act as small molecular receptor blockers, kinase inhibitors, protease inhibitors, pro-apoptotic factors, spindle poisons, DNA damaging agents and cell cycle inhibitors that can influence the modulation of signaling pathways in order to impede or cure cancer. RSV effects on NOTCH signaling pathways are unique in a way that, it causes activation rather than inhibition of different proteins of NOTCH signaling leading to its anti-cancer activity (Farooqi et al., 2018). RSV has been shown to be affecting diverse cancer stemness signaling pathways that control not only the cancer stemness but also other cancer properties like cell viability, proliferation, apoptosis induction, inhibiting cell migration, etc. as reported by different researchers in various model systems enlisted in Table – 2.

**Table 2:** Summary of RSV impact on cancer stem cell signaling pathways reported.

Signaling Pathway	Experimental Model Systems	RSV Effects	Ref.
Notch signaling	Glioblastoma cell lines (A172 and T98G)	Notch-1 activation **↑** dependent p53 mediated anti-proliferative and pro-apoptotic effects	Lin et al. (2011)
	Human GI carcinoid tumor cell lines (BON); Human pulmonary carcinoid cell lines (NC–H727)	Growth **↓** through S-phase cell cycle arrest Expression of neuroendocrine (NE) peptides/hormones chromogranin A and serotonin through activation of the Notch-2 isoform **↑**	Pinchot et al. (2011)
	Anaplastic Thyroid Carcinoma (ATC) Cell Lines (HTh7 and 8505C)	Dose-dependent inhibited ATC growth **↓** Cell differentiation **↑** via activation of Notch-1 signaling **↑**	Yu et al. (2013)
Wnt signaling	Colorectal cancer cell lines (LoVo cells)	Dose-dependent inhibition of the nuclear localization of β-catenin **↓** c-Myc and MMP-7 **↓** Cell proliferation and invasion **↓** These effects of RSV are opposite to that of the long non-coding RNA-MALAT1 cell proliferation and invasion abilities	Ji et al. (2013)
	Human normal breast epithelial cell line (MCF10A) and breast cancer cell line (MCF-7, SUM159)	Wnt/β-catenin pathway proteins **↓** β-catenin **↑** markedly reduced RSV-induced cytotoxicity and autophagy	Fu et al. (2014)
	Human normal (CCD112CoN) and colorectal cancer cell lines (HCT116, SW480, LoVo and CaCo-2)	TCF4 transcription factor expression **↓** via wnt/β-catenin pathway Phosphorylation of TCF4 **↑**via ERK and P38 dependent pathways Apoptosis **↑**	Jeong et al. (2015)
	Glioblastoma patient derived stem cells (GBM2, GBM7, G144, G179, G166, GliNS2, GBM04)	Wnt and EMT activator mediated GSC cell proliferation **↓** Cell mortality **↑** Cell motility**↑**	Cilibrasi et al. (2017)
	Squamous cell carcinoma cell line (Colo 16 cells)	RSV (100 μM) exhibited Wnt **↓** leading to Cell growth **↓** Apoptosis **↑** Transfection with β-catenin-specific siRNA enhanced RSV susceptibility	Liu et al. (2017)
	GC-MSCs derived from the gastric adenocarcinoma patient tissues	RSV reversed the progress of EMT Metastasis **↓** Wnt/β-catenin pathway proteins **↓**	Yin et al. (2020)
SHH signaling (Sonic Hedgehog Pathway)	Chronic myeloid leukemia cells (K562 cells)	RSV acted as Bcr-Abl inhibitor SHH pathway proteins **↓** patched (PTCH) **↓** Smoothened (Smo) **↓** Gli-1 **↓** Viability of CML cells **↓**	Liao et al. (2012)
	Acute Myeloid Leukemia (AML) patient derived mononuclear cells (MNCs).	RSV blocked IL-6 stimulated growth of AML cells through SHH signaling	Su et al. (2013)
	Human colorectal cancer cell lines (HCT116 cells)	Cell viability and migration **↓** Apoptosis **↑** SHH pathway proteins **↓**	Du et al. (2016)
	Renal cancer stem cells (ACHN and 786-O cells)	Size and number of tumorspheres **↓** via SHH signaling While purmorphamine up regulated SHH pathway and weakened the RSV effects	Sun et al. (2020)
PI3K Signaling	Human colon cancer cells (HCT116 cells)	Anti-proliferative effects **↑** via PTEN/PI3K/Akt and Wnt/β-catenin pathway protein regulation	Liu et al. (2014)
	Glioblastoma patient derived Glioblastoma-initiating cells (GICs)	Invasion and migration of GICs **↓** via suppressing PI3K/Akt/NF-κB and MMP-2 expression **↓**	Jiao et al. (2015)
	Adriamycin resistant chronic myeloid leukemia cell line (K562/Adr)	Anti-proliferative activities of bestatin **↑** P-gp expression **↓** via PI3K/Akt/mTOR signaling pathway	Wang et al. (2016)
	Human colorectal cancer cell lines (HCT116 cells)	Anti-cancer activity **↑** PI3K/AKT signaling **↓** BMP7 **↑** Phosphorylation of Akt1/2/3 and PTEN **↑**	Zeng et al. (2017)
	Human promyelocytic leukemia cells (HL-60) and ADR (Adriamycin)-resistant cell line (HL-60/ADR)	Drug resistance **↓** via PI3K/AKT/Nrf2 signaling and MRP1 expression	Li et al. (2019)
	Human acute promyelocytic leukemia cell lines (NB-4 and HL-60 cells)	PTEN expression **↑** PI3K/AKT pathway proteins **↓** Cell proliferation **↓** Apoptosis **↑**	Meng et al. (2019)
	Human small-cell lung cancer cell lines (H446 cells)	Cell viability **↓** and apoptosis **↑**via PI3K/Akt/c-Myc pathway	Li et al. (2020)
	Human papillary thyroid cancer cell lines (KTC-1 and TPC-1 cells); Mouse xenograft models	Anti-tumor effects **↑**of rapamycin mediated by PI3K/AKT/mTOR pathway	Bian et al. (2020)
	Murine melanoma cell line (B16–F10), human melanoma cell line (A375)	AKT/mTOR pathway proteins **↓** Autophagy **↑** Growth, viability and migration **↓**	Gong et al. (2020)
TGF/SMAD Signaling	Human epidermoid carcinoma cell lines (A431) and mouse models	Ultraviolet B (UVB) induced malignant tumor progression **↓** in p53+/−/SKH-1 mice through Akt mediated TGF-β2 **↓**	Kim et al. (2011)
	Colorectal cancer cell lines (LoVo cells)	Epithelial to mesenchymal transition (EMT) **↓** TGF-β1/SMAD signaling pathway **↓**	Ji et al. (2015)
	Human breast cancer cell lines (MDA-MB-231) and xenograft mouse model	Migration and metastasis **↓** by reversing TGF-β1 induced EMT	Sun et al. (2019)
	Human glioblastoma multiforme cell lines (LN18, U87 cells)	EMT **↓** EMT-generated stem cell like properties **↓** via Smad-dependent signaling regulation	Song et al. (2019)
NF-κB Signaling	Human multiple myeloma cell lines (U266), Patient derived MM.1 or MM.1S cells	Constitutive and IL-6 induced activation of STAT3 **↓** Constitutive activation of NF-κB **↓** Cell proliferation **↓** Sensitization of bortezomib and thalidomide mediated apoptosis **↑**	Bharadwaj et al. (2007)
PPAR pathway	Human colon carcinoma cell lines (SW480, HCT116, Caco2 and SW620)	Apoptosis **↑** Cell proliferation **↓** in combination with PPARγ	Aires et al. (2014)
	Bovine arterial endothelial cells (BAECs) and PPARα knockout mice	RSV exerted agonistic activity of PPARα as its direct target mediating long term effects of RSV under *in vivo* conditions	Takizawa et al. (2015)
JAK/STAT Pathway	Medulloblastoma cell lines (UW228-2 and UW228-3 Cells)	Bcl-2 expression **↑** STAT3 **↓** Survivin, cyclin D1, Cox-2 and c-Myc **↓** Growth suppression **↑** Differentiation-like changes **↑**	Yu et al. (2008)
	Human osteosarcoma cell lines (MNNG/HOS, MG-63 cells), osteoblast cell line (hFOB1.19 cells)	Cell proliferation and tumorigenesis **↓** correlated with cytokines inhibition related JAK2/STAT3 signaling blockage	Peng et al. (2018)
	Human ovarian cancer cell lines (SKOV3, Caov-3, OVCAR-4 and OVCAR-8 Cells)	RSV analog – pterostilbene exhibited anti-tumor activity via anti-proliferative and pro-apoptotic mechanisms through JAK/STAT3 pathway **↓**	Wen et al. (2018)

### 3.5 Resveratrol effects in combination with other molecules

The cell environment is a multi-factorial system and biologically active phytochemicals in its isolation shows differential effects due to the possible lack of secondary metabolite interaction with other molecules. Further, drug targeting by multiple strategies is one of the effective treatment regimens in cancer therapy and management, to come over the multi-drug resistance (MDR). Based on these facts, RSV treatment strategy was used in combination chemoprevention with other natural active molecules or small molecular drugs by several researchers to find the improved efficacy of RSV action. Pace-Asciak et al. (1995) reported that trans-resveratrol and quercetin combination present in red wine has shown to exhibit dose-dependent inhibition of both thrombin-induced and ADP-induced platelet aggregation preventing atherosclerosis more effectively. Initially RSV was found to be a potent anti-oxidant molecule that can prevent carcinogenesis, later several reports have indicated that it can mediate its actions through multiple ways by interacting with several molecules. Quercetin is another phytoconstituent that has been widely distributed in vegetables and fruits with many health enhancing effects along with anti-cancer effects like loss of cancer cell viability, inducing apoptosis and autophagy through PI3K, Wnt and MAPK pathway modulation (Anand David et al., 2016; Reyes-Farias and Carrasco-Pozo, 2019). Nam et al. (2016) have mentioned the various methods for nanofabrication of quercetin formulations and its applications in oncotherapy. The first combination of RSV tested was with quercetin on oral cancer cell growth and proliferation. It was reported that by treating with 50 μM RSV along with 10, 25 and 50 μM of quercetin which is another natural active component in common foods, oral squamous carcinoma cell (SCC-25) resulted in gradual significant increase in the inhibitory effect of quercetin on cell growth and DNA synthesis. Effective inhibition of SCC-25 cell growth and proliferation was reported due to enhanced activity of quercetin in presence of RSV (ElAttar and Virji, 1999). Combinational chemoprevention is only possible strategy to manage cancer cells and the same could be tested to target cancer stem cells. Different reports using various cancer and CSC models treated with RSV in combinations and their effects are presented in Table 3.

**Table 3:** Summary of RSV and its combinational chemopreventive effects.

Ref.	Experimental Models Systems	RSV Combinations	Effects
Bader & Getoff (2006)	Human Breast cancer cells – MCF7	Mitomycin C (MMC)	Anti-tumor free radical scavenger activity under aerobic conditions in presence of mitomycin **↑**
Reiter et al. (2007)	Human Mast Cell line-1 (HMC-1)	Delta-Tocopherol	*Combinations of* 50 μM *RSV and* 50 μM *delta-tocopherol resulted:* Protein Kinase B (PKB) Ser473-phosphorylation **↓** HMC-1 cell proliferation **↓**
Zhang et al. (2014)	Fanconi anemia (FA) murine models	N-acetylcysteine (NAC)	Neither RSV nor NAC could have significant chemopreventive effect in FA mouse models.
Yang et al. (2015a, b, c)	Human bronchial epithelial cell line BEAS-2B, 16HBE and Human lung cancer cell lines – A549 and H446.	AK001796 lncRNA	*AK001796 in lung cancer tissues and cells pretreated with RSV resulted:* G0/G1 cell cycle arrest **↑** In vitro and In vivo colony formation **↓** Cell growth and proliferation **↓**
Li et al. (2016)	Patient derived glioblastoma-initiating cells.	Temozolamide	*Both in vitro and in vivo resulted:* Apoptosis **↑** through DNA double stranded breaks, pATM/pATR/p53 pathway activation **↑** cell differentiation **↑** p-STAT3 activity **↓**
Hardin et al. (2016)	Anaplastic thyroid cancer cell lines – FRO, Kat18, NTHY-Ori-3, 8505C, papillary thyroid carcinoma cell line BCPAP, TPC-1 Cell line, THJ-16T and THJ-21T	Valproic acid	Stem cell marker - Aldefluor expression **↓** Proliferation **↓** Invasiveness **↓** Apoptosis **↑** Thyroid differentiation markers **↑**
Yuan et al. (2017)	Human ovarian carcinoma cell line – A2780 cells	Gemcitabine (GEM) along with Silver nanoparticles-RSV (AgNPs)	Combined GEM and AgNPs exhibited potent apoptotic activity **↑**
Dewangan et al. (2017)	Human breast cancer cells (HBCCs) - MCF-7, MCF-10A	Salinomycin	Apoptosis **↑** via reactive oxygen species (ROS) mediated mitochondrial dysfunction. Altered nuclear morphology PARP cleavage **↑**, Caspase activation **↑** Modulated MAPK pathway
Mukherjee et al. (2018)	C57BL/6 male mice (2–4 months old); GL261 mouse glioblastoma cells	TriCurin: Curcumin, Epicatechin gallate and Resveratrol (4:1:12.5) combination	*In GL261 under In vitro:* p53 **↑** apoptosis **↑** *In In vivo:* Repolarization of M2-like tumor (GBM) associated microglia/macrophages to the tumoricidal M1-like phenotype and intra-GBM recruitment of activated natural killer cells leading to apoptosis of tumor stem cells.
Pouyafar et al. (2019)	SKOV3 derived ovarian cancer stem cells *In vitro*	Doxorubicin (DOX)	*Treated with RSV and DOX at IC*_*50*_*of* 55 μM *and 25 ηM, respectively resulted:* BAX **↑** Caspase 3 **↑** MDR1 **↓** MRP1**↓** Drug resistance to doxorubicin **↓** Apoptosis **↑**
Pouyafar et al. (2019)	Cancer stem cells of human adenocarcinoma cell line HT-29	Sulindac	*Transcription of autophagy signaling genes*: (GALNT11) **↑** in cancer stem cells Trans-differentiation **↑** Decreased cell resistance **↓**
Hoca et al. (2020)	PANC-1 derived CD133+ and CD133- pancreatic cancer cells	Quercetin	*At 5,10,25,50 and* 100 μM *concentrations of combined treatment of CD133+ cells resulted:* ACTA-2, IL-1β, and N-Cadherin **↓** TNF-α and Vimentin **↑** TNF-α and N-Cadherin **↓** in RSV alone treated CD133+ cells Quercetin could prevent EMT to a greater extent than RSV
Shin et al. (2020)	HeLa cervical cancer adherent and stem-like cells	Pterostilbene	*Pterostilbene exhibited better effects than RSV including:* Cell cycle arrest at G2/M phase ROS-mediated Caspase-dependent apoptosis **↑** MMP-2/9 expression **↓** Tumor sphere formation and migration abilities **↓** *Stemness marker expression:* CD133, Oct-4, Sox2, and Nanog **↓** STAT-3 **↓**

### 3.6 Limitations of resveratrol in therapy

The critical point of limitation found in RSV literature is lack of sufficient *in vivo* and human trial based evidences. Its observations are mainly limited due to its bioavailability under *in vivo* system and also due to differential effects with different RSV concentrations. Further, there is a need for clear understanding for the roles of RSV metabolites along with free form of RSV as chemotherapeutic in cancer patients. Zykova et al. (2008) reported that in human colon adenocarcinoma HT-29 cells shown inhibited Cox-1 and Cox-2 by both RSV and its metabolite RSV-4′-O-sulfate. In another study, the hydroxylated metabolites of RSV formed from gut microbiota have exerted cytotoxic properties (Bode et al., 2013). These effects in other type of cancers and in clinical studies require proper validation.

It was established for RSV effects like NF-κB activity regulation, inhibiting cytochrome P450 isoenzyme (CYP A1), cyclooxygenase (COX) activity, TP53, FAS/FASL or CD95 induced apoptosis, inhibiting the HIF-1α and VEGF expression through which its anti-cancer properties are sought. There are few clinical trials with RSV as oral administration or micronized formulations for different type of cancer patients. Few studies have indicated its advantageous effect by modulating the targeted molecules, few were inconclusive and other few studies have resulted with certain adverse effects like nausea, diarrhea, vomiting, fatigue, anemia and mainly renal toxicity in multiple myeloma patients (Popat et al., 2001).

There are controversial reports which need to be reconfirmed and studied in details. RSV was reported to promote atherosclerosis in hypercholesterolemic rabbits rather than protecting against atherosclerosis (Wilson et al., 1996). Further, RSV was shown to suppress atherosclerosis in hypercholesterolemic rabbits without affecting plasma lipid levels (Wang et al., 2005). Zhang et al. (2014) reported that tempol and N-acetylcysteine (NAC) or RSV when tested for its chemopreventive effects in tumor prone Fancd2(−/−)/Trp53(±) fanconi anemia (FA) murine models, RSV could not show effective chemopreventive effect as that of tempol. There are certain clinical trials attempted to draw conclusions for RSV as an effective chemotherapeutic is discussed in the review by Berman et al. (2017) and they reported that breast cancer and multiple myeloma patients have shown RSV as more promising molecule but limited due to adverse effects. Other clinical trials were made on prostate cancer, colorectal cancer and bladder cancer patients, but require further detailed understanding of RSV effects.

## 4 Conclusions and perspectives

Natural bioactive compounds in edibles with pharmacological activities have no known side effects and can have better impact by interacting with other secondary metabolites. Hence, at present potent natural bioactive compounds and their applications are on demand. RSV is a well-known compound and recently its effects of targeting CSCs have become more interesting. Being a potent reducing agent it is known to prevent carcinogenesis due to its anti-oxidant abilities, however its ability to regulate other molecules and mechanisms to target cancer cells and cancer stem cells are now attaining interest.

After the initial report in 2007, in which RSV was reported to stop cell shedding, thus inhibiting metastasis of mouse cancerous mammospheroid cells, following research on CSCs have tremendously taken a peak with most of the research groups working either with RSV alone or in combination with other molecules to test anti-cancer stem cell effects. There has been intervention of recent methods like effect of RSV and long noncoding RNAs (lncRNAs) in lung carcinogenesis (Yang et al., 2015a, b, c), inhalable resveratrol-cyclodextrin complex loaded biodegradable nanoparticles against non-small cell lung cancer (Wang et al., 2020) and as an immunomodulatory agent (Trung and An, 2018) in immunotherapy of treating cancer and cancer stem cells are some areas at the front end of modern research.

The major drawback of RSV research is that, most of the attempts include *in vitro* cell culture experiments that require validation of the same effect under *in vivo* conditions and with primary cultures of human cancer tissues along with clinical trials. As RSV is a natural bioactive compound, it should be tested with different combinations as they can affect multiple pathways unlike targeted drug molecules which make this strategy as unique therapeutic regiment to target cancer cells and cancer stem cells.

## CRediT author statement

All authors contribute to Conceptualization, Methodology, Investigation, Writing- Original draft preparation, and Writing- Reviewing and Editing.

## Declaration of competing interest

The authors declare that they have no known competing financial interests or personal relationships that could have appeared to influence the work reported in this paper.
